# Genotyping MUltiplexed-Sequencing of CRISPR-Localized Editing (GMUSCLE): An Experimental and Computational Approach for Analyzing CRISPR-Edited Cells

**DOI:** 10.1089/crispr.2023.0021

**Published:** 2023-10-10

**Authors:** Peng Zhang, Laurent Abel, Jean-Laurent Casanova, Rui Yang

**Affiliations:** ^1^ St. Giles Laboratory of Human Genetics of Infectious Diseases, Rockefeller Branch, The Rockefeller University, New York, New York, USA; Necker Hospital for Sick Children, Paris, France.; ^2^ Laboratory of Human Genetics of Infectious Diseases, Necker Branch, INSERM UMR1163, Paris, France; Necker Hospital for Sick Children, Paris, France.; ^3^ University Paris Cité, Imagine Institute, Paris, France; Necker Hospital for Sick Children, Paris, France.; ^4^ Howard Hughes Medical Institute, New York, New York, USA; and Necker Hospital for Sick Children, Paris, France.; ^5^ Department of Pediatrics, Necker Hospital for Sick Children, Paris, France.

## Abstract

Clustered regularly interspaced short palindromic repeats (CRISPR)-CRISPR-associated protein 9 (Cas9) creates double-stranded breaks, the repair of which generates indels around the target sites. These repairs can be mono-/multi-allelic, and the editing is often random and sometimes prolonged, resulting in considerable intercellular heterogeneity. The genotyping of CRISPR-Cas9-edited cells is challenging and the traditional genotyping methods are laborious. We introduce here a streamlined experimental and computational protocol for genotyping CRISPR-Cas9 genome-edited cells including cost-effective multiplexed sequencing and the software Genotyping MUltiplexed-Sequencing of CRISPR-Localized Editing (GMUSCLE). In this approach, CRISPR-Cas9-edited products are sequenced in great depth, then GMUSCLE quantitatively and qualitatively identifies the genotypes, which enable the selection and investigation of cell clones with genotypes of interest. We validate the protocol and software by performing CRISPR-Cas9-mediated disruption on interferon-α/β receptor alpha, multiplexed sequencing, and identifying the genotypes simultaneously for 20 cell clones. Besides the multiplexed sequencing ability of this protocol, GMUSCLE is also applicable for the sequencing data from bulk cell populations. GMUSCLE is publicly available at our HGIDSOFT server and GitHub.

## Introduction

The Cas9 protein (CRISPR-associated protein 9), an RNA-guided DNA endonuclease derived from the type II CRISPR (clustered regularly interspaced short palindromic repeats) of bacteria, is a powerful tool for targeted engineering of the genome in diverse organisms.^1^ The Cas9 DNA endonuclease can be guided to target loci by a stretch of chimeric RNA, known as single-guide RNA or sgRNA.^2,3^ At the target locus, Cas9 edits the gene by creating double-stranded breaks, which are then repaired by host-cell DNA repair mechanisms. Nonhomologous end joining (NHEJ) results in random indels and gene disruption at the targeted loci (knockout), whereas homology-directed repair in the presence of a DNA template can result in the knock-in of a specific sequence of interest.^3–6^ CRISPR-Cas9-based gene disruption is flexible and easy to use, with a high editing efficiency, and this method can be used to target almost all sites of interest in genomes.

However, accurate genotyping of the targeted loci has proved challenging, for several reasons. First, NHEJ is random and generally results in short indels of highly variable sizes.^7^ Second, editing may be mono-allelic or multi-allelic, rendering targeted sequencing inappropriate due to the resultant sequence heterogeneity. Third, some widely used CRISPR-Cas9 delivery systems, such as the pLenti-CRISPR-V2 plasmid,^8^ result in the constitutive expression of Cas9 and sgRNA, which may edit the targeted loci more than once, thereby creating a higher inter-cellular heterogeneity even within single-cell clones. Many groups have tried to genotype their CRISPR-Cas9-edited products by subcloning target loci in competent bacteria and then performing colony sequencing (PCR and Sanger sequencing),^9^ or by measuring protein expression levels,^10,11^ both of which are time-consuming and inefficient, whereas sometimes the genotyping is skipped.

Several tools have been developed for the assessment of gene-editing quality or determination of mutational spectrum of CRISPR-Cas9 experiments, by analyzing massively parallel sequencing data, also known as next-generation sequencing data, from gene-edited cells. These tools include CLiCKAR,^12^ CRISPResso2,^13^ CrispRVariants,^14^ Cas-analyzer,^15^ and CRISPR-GA.^16^ However, these tools have several limitations, which drove our attempts to develop another effective tool for this purpose. CLiCKAR is provided as a webserver and can be used for multiplexed sequencing for genotyping CRISPR-Cas9-knockout samples.^12^ However, it does not work for demultiplexed sequencing data, even though demultiplexing is often a standard build-in procedure in sequencing platforms; it only accepts single-end data; it excludes deletions >40 nucleotides; and one mandatory input (query_table.csv) is not well described. CRISPResso2 is provided as a command-line tool and a webserver, and is particularly useful for base-editing assays, with a detailed output of nucleotide substitutions. Its command-line tool operates on Bioconda system, which could be very difficult for most of the scientists studying gene-editing. CrispRVariants works only with BAM files, and requires R programming skills. Cas-analyzer and CRISPR-GA require users to upload one sample at a time through their websites, making them unsuitable for studying large-scale samples. A common issue of all these tools is that they provide genotypes as sequence alignments, not in VCF format (CHR-POS-REF-ALT) which is conventionally used for representing variants and genotypes, for which it requires users' additional effort to identify the genotypes manually and individually. We therefore aimed to develop a streamlined experimental and computational approach for genotyping of CRISPR-edited cells that was precise, efficient, user-friendly, and affordable to facilitate the genome-editing studies.

## Materials and Methods

### Sample preparation

Two sets of single-stranded oligonucleotides were designed to generate double-stranded duplexes for pLenti-CRISPR-V2 construction. In brief, forward 5′-CAC CGG TAC ATT GTA TAA AGA CCA C-3′ and reverse 5′-aaa cGT GGT CTT TAT ACA ATG TAC c-3′ strands were ordered in pairs (Integrated DNA Technologies). Forward 5′-CAC CGT TGT ATA AAG ACC ACA GGT A-3′ and reverse 5′-aaa cTA CCT GTG GTC TTT ATA CAA c-3′ strands were also ordered in pairs (Integrated DNA Technologies). Each pair of oligonucleotides was annealed and inserted into pLenti-CRISPR-V2 constructs as previously described.^8^ Both gRNA sequences were designed to target the 3′ end of exon 5 of interferon-α/β receptor alpha (*IFNAR1*). Culture supernatants containing lentiviruses targeting *IFNAR1* were produced in HEK 293T cells transfected with constructs of interest. Lentivirus-containing supernatants were concentrated by a factor of 10, with Lenti-X (Takara Bio).

### CRISPR-Cas9 editing

HEK 293T and HeLa (ATCC CCL-2 line) cells were plated in 12-well tissue-culture plates. Lentiviruses containing both pLenti-CRISPR-V2 constructs targeting *IFNAR1* were added to cultured HEK 293T and HeLa cells. We added 1 μg/mL puromycin to cell cultures to ensure the selection of puromycin-resistant cells. Puromycin-resistant HEK 293T and HeLa were harvested and serially diluted to 3 cells/mL. We plated 200 μL of cell suspension in each well of 96-well tissue-culture plates at an estimated density of 0.6 cells/well, for single-cell plating. Two weeks later, wells with grossly visible cell growth, identified by a change in culture medium color, were treated with trypsin and used to consolidate new sets of tissue-culture plates for expansion. Small numbers of cells were harvested for PCR genotyping.

In brief, genomic DNA (gDNA) from these single-cell clones was extracted with the Phire tissue direct PCR kit (Thermo Fisher Scientific). Three-primer competitive PCR was performed to genotype 72 single-cell clones derived from HEK 293T cells and 48 HeLA single-cell clones. The primers used for the three-primer competitive PCR were the forward-out primer: gttatctggaaaaactcttcaggtg, the forward-in primer: ctatagtccagtacattgtataaagaccac, and the reverse-out primer: gcccagcctgaacttttaagtag, as previously described.^17^ Briefly, the forward-in primer overlapped with the Cas9 cleavage site. Forward-out and reverse-out primers flanked the Cas9 cleavage site.

Single-cell clones with only one upper-band PCR product on agarose gel electrophoresis correspond to complete disruptions of all targeted *IFNAR1* alleles, with this sequence disruption preventing forward-in primer binding. Single-cell clones with only one lower-band PCR product probably correspond to unedited or insufficiently edited clones in which shorter amplicons formed by forward-in and reverse-out primers were preferentially amplified. Thus, single-cell clones with both upper and lower PCR bands probably correspond to clones that contain both insufficiently edited alleles and completely disrupted alleles. Ten single-cell clones each of HEK 293T cells and HeLa cells displaying probable complete disruption of all alleles were selected for multiplexed sequencing.

### Multiplexed sequencing

gDNA from these 20 single-cell clones was extracted with the Phire tissue direct PCR kit (Thermo Fisher Scientific). The first PCR (1st PCR), using the gDNA from each of these 20 clones, was performed with the same forward and reserve primers containing 5′ overhangs covering Tn5-transposase sequences. Specifically, we used the forward primer 5′-GCGTCAGATGTGTATAAGAGACAGgttatctggaaaaactcttcaggtg-3′ and the reverse primer 5′-GCTCGGAGATGTgtataagagacaggcccagcctgaacttttaagtag-3′. We then used 1 μL of 1st PCR products for each clone in a second round of PCR (2nd PCR) with the same forward primer Ad1_noMx: 5′-AAT GAT ACG GCG ACC ACC GAG ATC TAC ACT CGT CGG CAG CGT CAG ATG TG-3′, and a clone-specific indexed reverse primer 5′-CAA GCA GAA GAC GGC ATA CGA GAT i7index (NNNNNNNN) G TCT CGT GGG CTC GGA GAT GT-3′. The PCR products from the 2nd PCR were, therefore, individually barcoded.

These indexed PCR products were pooled and extracted by agarose gel electrophoresis (Qiagen). The sequences of all 24 indexed reverse primers ordered are shown in Supplementary Table S1.^18^ The pool of 20 indexed PCR products was sequenced with the Nano MiSeq 300bp system. Theoretically, paired-end sequencing would provide an optimal coverage of the regions of interest. In this experiment, 25 cycles were used for MiSeq Nextera Read 1 to pass the quality control step, with the remaining cycles used for MiSeq Nextera Read 2 reverse sequencing and i7 index sequencing.

### GMUSCLE input, design, and output

To use Genotyping MUltiplexed-Sequencing of CRISPR-Localized Editing (GMUSCLE) command-line tool, the user needs to provide the reference genome sequence, the genomic region of interest (CHR, START, END) that spans the CRISPR target site and is used for multiplexed sequencing, and the directory of the demultiplexed individual fastq files. To use GMUSCLE webserver, the user needs to choose the human reference genome, type in the genomic region of interest, and upload fastq data of one or multiple samples.

GMUSCLE works with both single-end and paired-end fastq data, and it uses fastq-join to preprocess paired-end data. GMUSCLE extracts the reference genome sequence of the given region and creates a BLAST database.^19^ For each individual's sequencing data, GMUSCLE merges all raw reads on the basis of their uniqueness and counts the occurrence of each unique read. The unique reads reaching the read-count cutoff (default value: 30) are retained for BLAST analysis against the reference genome sequence, with a configuration allowing gap opening and extension.^19^ GMUSCLE identifies indels and their supporting reads, and then outputs the indels supported by ≥1% of total raw reads as the major genotypes. Finally, GMUSCLE produces (1) a summary file; (2) a plot of the major genotypes and their proportions; (3) a sequence alignment of the major genotypes against wild-type (WT) sequence; and (4) a matrix of the major genotypes in all samples.

## Results

### An overview of the method

**FIG. 1.**
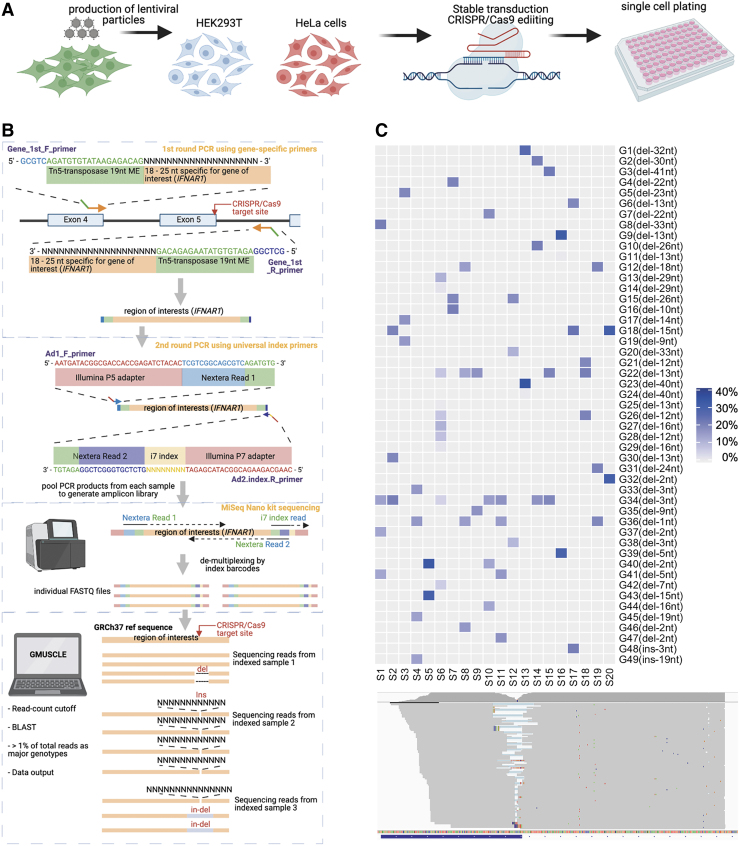
Schematic description of multiplexed sequencing and genotyping of CRISPR-edited cells by GMUSCLE. Schematic flow of **(A)** CRISPR editing, **(B)** multiplexed sequencing, and **(C)** the detection of major genotypes in the 20 samples, and the proportions of reads supporting these major genotypes (genotype details in Supplementary Tables S3 and S4). CRISPR, clustered regularly interspaced short palindromic repeats; GMUSCLE, Genotyping MUltiplexed-Sequencing of CRISPR-Localized Editing.

Our proposed protocol includes three major steps: (1) the CRISPR-Cas9 gene editing of targeted loci of interest; (2) amplification of the gDNA sequence spanning the target site and clone-specific barcoding, followed by multiplexed sequencing; and (3) the use of GMUSCLE software to analyze the demultiplexed sequencing data for simultaneous identification of the genotypes generated by the gene-editing of each cell clone (Fig. 1). In this article, we use pLenti-CRISPR-V2 system with lentiviruses to achieve CRISPR-Cas9 gene-editing,^8^ as the lentiviral approach is suitable for the transduction of both cell lines and primary cells. Single-cell plating and cloning are then performed to obtain a highly homogeneous cell population with minimal confounder effects. The gDNA spanning the target site is next amplified by two rounds of PCR, the second of which adds clone-specific barcoding DNA sequences to the PCR products. These individually barcoded amplicons are sequenced and demultiplexed with a cost-effective sequencing platform.

**FIG. 2.**
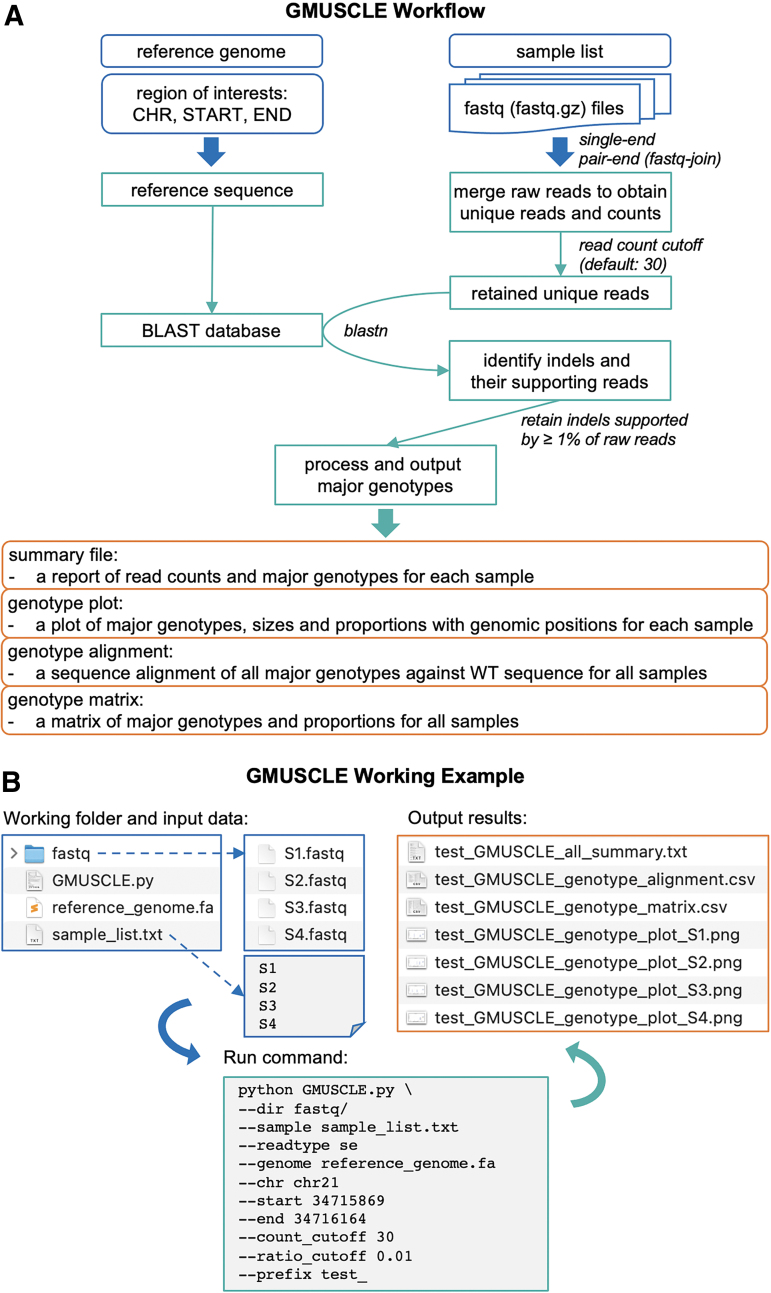
Illustration of **(A)** the workflow and **(B)** a working example of GMUSCLE software.

These individual sequencing data are then processed and analyzed by GMUSCLE, to provide (1) a summary file of the major genotypes (in VCF format) detected in each sample; (2) a plot of genotypes (insertions and deletions of different sizes, at different genomic positions) and their proportions in each sample; (3) a sequence alignment of all major genotypes against the WT sequence for all samples; and (4) a matrix of all genotypes and their proportions in all samples (Fig. 2A). This streamlined experimental and computational protocol, therefore, provides accurate genotype information for the edited cells, hence, facilitating the selection of certain cell clones with the genotypes of interest for further functional studies and investigations of genotype-phenotype associations.^10,20,21^ We will present below the method in more detail, by using a real example of editing the *IFNAR1* gene in 20 cell clones.

### CRISPR-Cas9 editing and multiplexed sequencing

We generated the IFNAR1-deficient HEK 293T and HeLa cells here as an example to illustrate the analysis protocol. The pLenti-CRISPR-V2 plasmids bearing sgRNAs targeting the 3′-end of exon 5 of *IFNAR1* were constructed.^8^ Lentiviral particles were produced, concentrated, and delivered to WT HEK 293T or HeLa cell lines (Fig. 1A). Sufficient disruption of the target region was confirmed, by a competition-based PCR method, for transduced HEK 293T and HeLa cell lines.^17^ Briefly, a three-primer PCR was used with the forward-in primer overlapping with the Cas9 cleavage site, while the forward-out and reverse-out primers flanking the Cas9 cleavage site. Single-cell clones with only one upper-band PCR product on agarose gel electrophoresis correspond to complete disruptions of all targeted *IFNAR1* alleles, with this sequence disruption preventing forward-in primer binding. Cell suspensions were serially diluted to obtain single-cell suspensions, which were plated in 96-well plates.

After incubation for 1–2 weeks, wells containing a grossly visible single colony were subjected to another competition PCR-based genotyping (Fig. 1A). We selected 10 single-cell clones from either HEK293T or HeLa cells, the *IFNAR1* loci of which were considered likely to be completely disrupted. We sequenced these single-cell clones by two-step PCR amplification (Fig. 1B). The gDNA extracted from each clone was amplified with *IFNAR1*-specific primers spanning the target site. We added 5′-overhang sequences to both the forward and reverse primers, to match a 19-nucleotide Tn5-transposome-specific mosaic end, followed by a short stretch of sequence complementary to the Nextera read primers. Individual PCR products from 20 clones were subjected to a second-round of PCR amplification with index primers previously used (see Materials and Methods section).^18,22^ These index primers, with each index barcode representing sequencing data from a single cell clone, were suitable for multiplexing and compatible with the Nextera Library Preparation system. The PCR products or amplicons were pooled, purified by gel extraction, and sequenced on the MiSeq platform with a 300 bp single-read Nano kit (which generates a total of 1 million reads for 20 samples, at a cost of ∼300 US dollars). Paired-end 250 bp-read sequencing is also available with the Nano Kit, making it possible to achieve a coverage of 500 bp. We used 25 cycles for MiSeq Nextera Read 1 to pass the quality control step, with the rest of the cycles used for MiSeq Nextera Read 2 reverse sequencing and i7 index sequencing. The sequence data were automatically demultiplexed into individual fastq files.

### GMUSCLE for identifying the genotypes from gene-editing

We used the human reference genome GRCh37, provided the genomic region of *IFNAR1* of interest (chr21: 34715869–34716164), and loaded fastq files of 20 samples (S1 to S20, with 10 cell clones from either HEK293T or HeLa cells) into GMUSCLE for identifying the genotypes present in these samples (Figs. 1C and 2B). The raw fastq data included a total of 50,000–70,000 sequencing reads for each clone, of which 10,000–15,000 reads are unique. Applying a read-count cutoff ≥30 for unique reads, we retained a subset of 30,000–40,000 reads (∼60% of total reads, but from less than 50 unique reads) for genotyping (Supplementary Fig. S1 and Supplementary Table S2). This cutoff can be set to zero to retain all reads for genotyping, if necessary. The retained reads were used to BLAST against the reference sequence in the genomic region of interest, with the configuration allowing gap opening and extension.^19^ GMUSCLE then identified all indels and their supporting reads, and outputted the indels that were supported by ≥1% of total reads as the major genotypes, for each sample.

In our 20 samples, a total of 49 major genotypes were identified. Each sample contained 2–4 major genotypes, except that S6 had 10 major genotypes. These 49 genotypes consisted of 47 deletions and 2 insertions, with deletions of 1–3 and 12–15 nucleotides being more frequent than indels of other sizes (Fig. 1C; Supplementary Tables S3 and S4). There were 32 genotypes from 10 HEK293T cell clones (S1–S10), 25 genotypes from 10 HeLa cell clones (S11–S20), with 8 overlapping genotypes. Chromosome 21 in most HEK293T cells is triploid or tetraploid.^23^ Similarly, it is mostly diploid, triploid, or tetraploid in HeLa cells.^24^ Notably, the numbers of major genotypes identified by GMUSCLE match the expected numbers of chromosome 21 in most corresponding cell clones. However, it is also possible that the presence of more than two genotypes in some clones are secondary to polyclonality as a result of inevitable contamination during single-cell plating step or genomic instability in cancer cell lines. The unusually high number of genotypes identified in S6 is probably due to the presence of multiple clones or prolonged Cas9-mediated disruption.

GMUSCLE also outputs a plot to visualize the major genotypes and their proportions at specific genomic positions for each sample (Supplementary Fig. S2), and a sequence alignment file to detail the mutational changes at nucleotide-level. GMUSCLE therefore provides an efficient and precise approach for the quantitative and qualitative analysis of genome outcomes from CRISPR-Cas9-editing experiments.

### The use of read-count cutoff to retain unique reads for genotyping, and the time cost

**FIG. 3.**
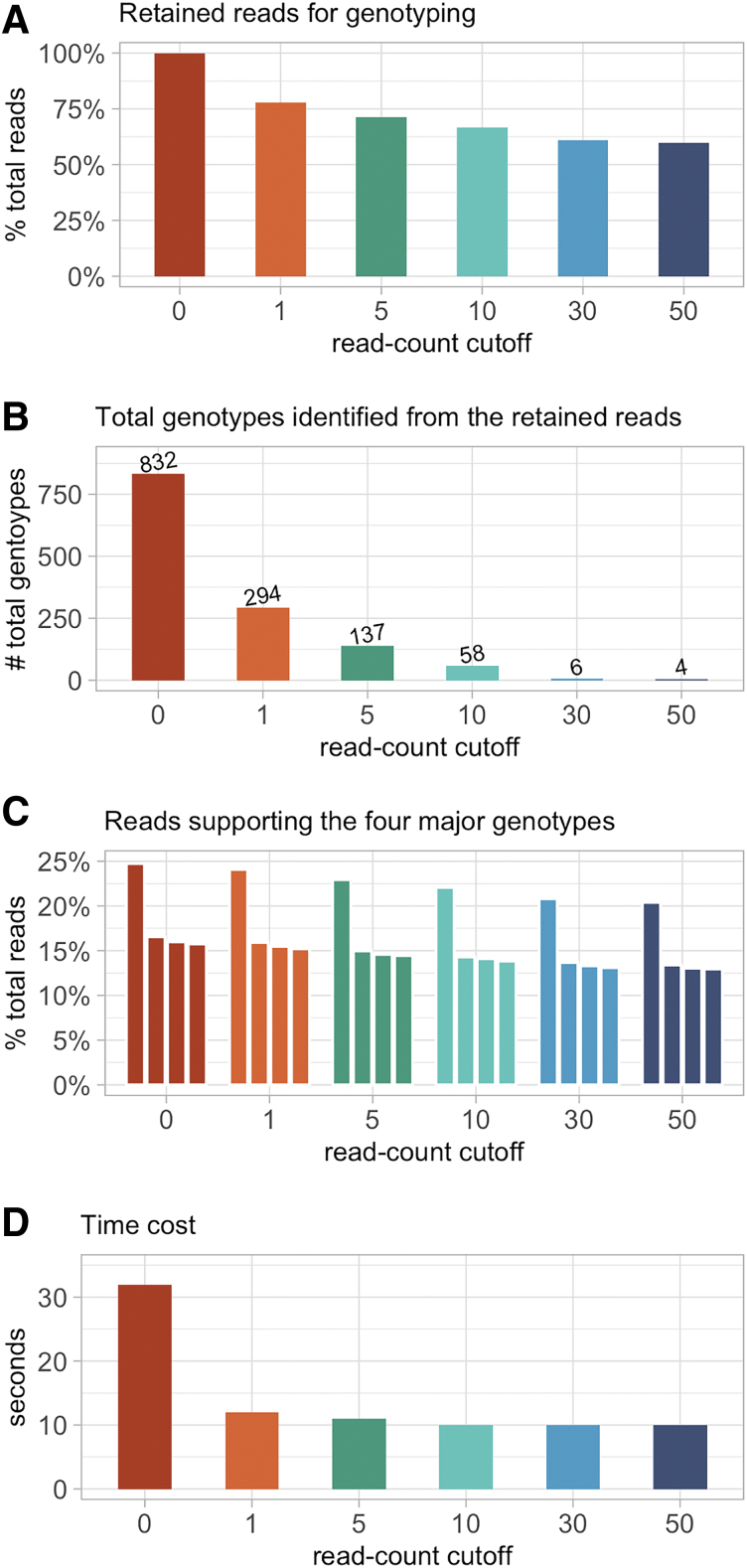
The performance and outcome of applying different read-count cutoffs for identifying the major genotypes, using sample S1 as an example. **(A)** percentage of total reads retained for genotyping, **(B)** total number of genotypes identified from the retained reads, **(C)** proportion of the retained reads supporting the four invariably major genotypes, and **(D)** time cost for different read-count cutoffs.

We validated the use of read-count cutoff as a feasible way for reducing the number of raw fastq reads for genotyping, using sample S1 to demonstrate its performance and outcome (Fig. 3 and Supplementary Table S5). We first tested GMUSCLE with 100% of the 56,269 raw reads from S1 (read-count cutoff = 0), which included 14,336 unique reads. The analysis detected 832 genotypes, only 4 of which were identified as major genotypes on the basis of support by ≥1% of total reads (24.7%, 16.5%, 16.0%, and 15.8%, respectively). We measured a parameter “read contribution to major genotypes,” which was defined as the number of reads supporting all major genotypes divided by the number of reads retained for genotyping. This parameter had a value of 0.73 at the read-count cutoff of 0. We then tested the read-count cutoff at values of 1, 5, 10, 30, and 50, corresponding to the decreased proportions of raw reads retained for genotyping to 77.8%, 71.2%, 66.6%, 61.0%, and 59.8%, which lead to the output of 294, 137, 58, 6, and 4 genotypes, respectively (Supplementary Table S5). The four major genotypes, which were identified at read-count cutoff = 0, were invariably detected at read-count cutoffs from 1 to 50. When the read-count cutoffs were 30 and 50, the parameter “read contribution to major genotypes” reached ∼1, which implies that almost all the reads retained for genotyping belonged to the four major genotypes.

In terms of time cost, it took 32 s for a regular office desktop computer to complete the genotyping of S1 with read-count cutoff = 0, and ∼10 s for the other cutoffs (Fig. 3). The baseline time cost is due to the extraction of genomic sequences of interest from the reference genome. The use of read-count cutoff (default value: 30) did not affect the detection of major genotypes, but increased the utility of the retained reads for genotyping, eliminated the random mutations introduced by enzymes in CRISPR-Cas9 experiments, and reduced the time cost. In addition, we merged data of all 20 samples into one single fastq file (∼1.2M reads in total), to pretend it as a sequencing data from bulk cell populations, and GMUSCLE took 15 s to complete the genotyping.

### Comparison with other existing tools

Several tools (CLiCKAR,^12^ CRISPResso2,^13^ CrispRVariants,^14^ Cas-analyzer,^15^ and CRISPR-GA^16^) have been published for assessing the gene-editing quality or determining the mutational spectrum by analyzing sequencing data from CRISPR-Cas9 experiments. After some testing, we focused on comparisons of GMUSCLE with CRISPResso2 and Cas-analyzer. We excluded three tools (CLiCKAR, CrispRVariants, and CRISPR-GA), because CLiCKAR does not accept demultiplexed sequencing and one mandatory input was not well described; CrispRVariants is mainly for visualizing gene-edited sequence alignments; and the data uploading to CRISPR-GA was very slow, and it failed to return any results in our testing.

**FIG. 4.**
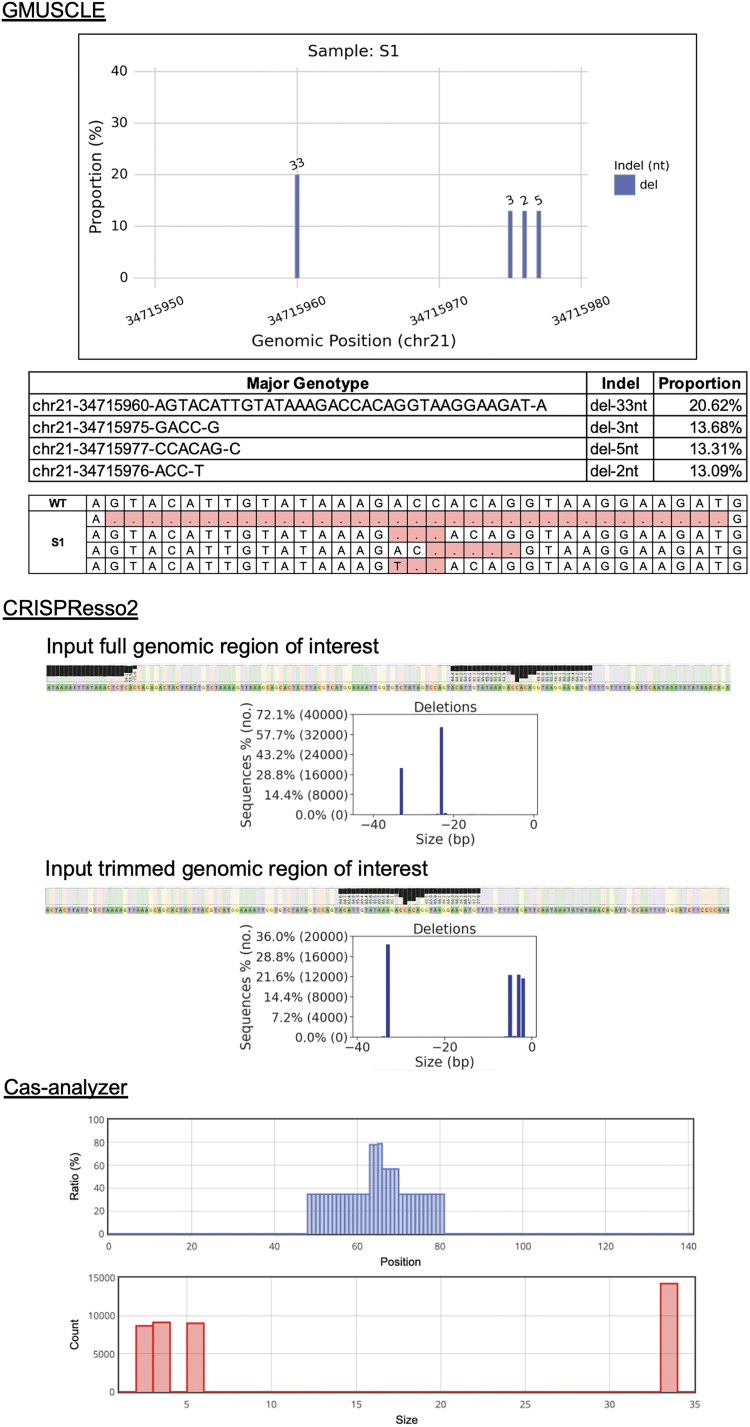
Comparison of GMUSCLE, CRISPResso2, and Cas-analyzer for the analysis of sample S1.

We used the 20 samples from our *IFNAR1*-editing experiment for CRISPResso2 and Cas-analyzer analyses, respectively (Fig. 4). In the study of CRISPResso2, we found it recognized the unaligned ends of sequencing reads as deletions, while no such problem occurred with GMUSCLE. We tried to resolve this problem by manually trimming the reference sequence as the input for CRISPResso2, which then led to GMUSCLE and CRISPResso2 generating identical genotypes for all 20 samples. In the study of Cas-analyzer, we found it difficult for large-scale studies, due to the need of uploading one file at a time through its website. Cas-analyzer identified identical genotypes as GMUSCLE for 12/20 samples, leaving eight samples with different outcomes (mostly missing deletions of 1–2 nucleotides). We tried to resolve this problem by removing its default parameter (WT marker), which then resulted in the identical genotypes for these eight samples. In addition, neither CRISPResso2 nor Cas-analyzer can provide genotypes in VCF format, which is conventionally used for representing variants and genotypes. CRISPResso2 provides a nucleotide frequency table, and Cas-analyzer provides a sequence alignment, both of which require users' additional effort to identify their cells' genotypes manually and individually.

## Discussion

The genotyping of CRISPR-Cas9-edited cells has proved challenging, and the traditional genotyping methods are often laborious and inefficient. As a result, many groups tend to skip the genotyping step and perform phenotyping or functional validation instead. Genotyping is superior to protein-level phenotyping or functional validation for several reasons. First, within any single-cell clone with some degree of heterogeneity introduced by constitutive editing or multi-clonality, protein-level phenotyping or functional validation could easily miss some proportions of cells carrying certain genotypes, such as in-frame deletion/insertion or missense mutation, that could still be functional. For genes of interest are involved in cell survival or cell cycle regulation, these missed cells may have growth advantage or even change the cellular phenotype. Second, if the region selectively targeted by CRISPR-Cas9 is within the epitope recognized by detection antibodies, the protein-level phenotyping ceases to be valid. In addition, as functional assays are not always available, genotyping may be the only possible approach. Finally, the knowledge of the major genotypes can guide the choice of cell clones suitable for specific experiments and enable investigators to subculture and study cells with specific genotypes of interest further.

With the recent trend of using sequencing data to assess gene-editing quality and to genotype gene-edited cells, causal genotype-phenotype relationships have been successfully identified in various organisms.^10,20,21^ We evaluated the existing tools for this purpose and identified several limitations that could be overcome. We therefore present here GMUSCLE, a convenient, precise, and efficient bioinformatics tool that is readily available to the scientific community to analyze CRISPR-edited cells. We also include a detailed bench protocol for *IFNAR1* gene depletion from two human cell lines, followed by multiplexed sequencing with the MiSeq Nano kit, as a demonstration of the first-time use of GMUSCLE.

GMUSCLE has several advantages: (1) it runs as a webserver with user-friendly interface, for users with less computational expertise and suitable for small-scale studies; it also runs by a single-line command with a few essential input parameters, for users with some computational experience and suitable for large-scale studies; (2) it outputs genotypes in VCF format, generates integrative and informative plots of major genotypes, and provides sequence alignment between edited genotypes and WT sequence at nucleotide-level; (3) it is fast and can process large-scale data in a single run, as in this study, it genotyped 20 samples with 50,000–70,000 sequencing reads each, within 2 min, using an office desktop computer; (4) it accepts both single-end and paired-end sequencing data and includes a built-in function for joining paired-end reads; and (5) it is resistant to random mutations outside the CRISPR-targeted sites introduced by PCR with Taq polymerase, as it focuses on the major genotypes (indels created by CRISPR-editing) supported by ≥1% of total reads. With these helpful features, GMUSCLE enhances the toolbox for genome-editing studies.

Our streamlined and integrated experimental and computational protocol will improve our approaches to the genotyping, screening, and study of CRISPR-Cas9-edited cells. In addition, besides the multiplexed-sequencing ability of this entire protocol, GMUSCLE software alone is also versatile. GMUSCLE is able to analyze the sequencing data: (1) from bulk cell populations in organs or tissues, for which the users just need to provide the large fastq files to GMUSCLE; (2) from cells that were treated with multiple sgRNA to edit multiple target sites, for which the users just need to run GMUSCLE multiple times with different genomic positions of the target sites; (3) from different experimental gene-editing protocols/systems, as GMUSCLE only needs sequencing data, reference genome, and genomic position from the users for its analysis; and (4) from gene-edited products in other organisms, for which the users only need to provide the organism's reference genome to run GMUSCLE. Therefore, GMUSCLE will be very useful to the scientific community studying molecular genetics and functional biology with CRISPR-Cas9 techniques.

## Software

GMUSCLE is publicly available at https://hgidsoft.rockefeller.edu/GMUSCLE and https://github.com/casanova-lab/.

## Supplementary Material

Supplemental data

Supplemental data

Supplemental data

Supplemental data

Supplemental data

Supplemental data

Supplemental data
