# Supplementary material for: Genotyping MUltiplexed-Sequencing of CRISPR-Localized Editing (GMUSCLE): An Experimental and Computational Approach for Analyzing CRISPR-Edited Cells
Source: CRISPR J. 2023 Oct 10;6(5):462–72. doi: 10.1089/crispr.2023.0021 (PMC10611965; doi:10.1089/crispr.2023.0021)
Supplement: Supplemental data [file Supp_TableS1.pdf]

Supplementary Tables

Table S1: Indexed reverse primers for multiplexed PCR amplification followed by multiplexed sequencing.

Well position	Sequence name	Sequence
A01	Ad1_noMX:	AATGATACGGCGACCACCGAGATCTACACTCGTCGGCAGCGTCAGATGTG
A02	Ad2.8_CAGAGAGG	CAAGCAGAAGACGGCATACGAGATCCTCTCTGGTCTCGTGGGCTCGGAGATGT
A03	Ad2.16_CCGTTTGT	CAAGCAGAAGACGGCATACGAGATACAAACGGGTCTCGTGGGCTCGGAGATGT
A04	Ad2.24_CCACTCCT	CAAGCAGAAGACGGCATACGAGATAGGAGTGGGTCTCGTGGGCTCGGAGATGT
B01	Ad2.1_TAAGGCGA	CAAGCAGAAGACGGCATACGAGATTCGCCTTAGTCTCGTGGGCTCGGAGATGT
B02	Ad2.9_GCTACGCT	CAAGCAGAAGACGGCATACGAGATAGCGTAGCGTCTCGTGGGCTCGGAGATGT
B03	Ad2.17_TGCTGGGT	CAAGCAGAAGACGGCATACGAGATACCCAGCAGTCTCGTGGGCTCGGAGATGT
C01	Ad2.2_CGTACTAG	CAAGCAGAAGACGGCATACGAGATCTAGTACGGTCTCGTGGGCTCGGAGATGT
C02	Ad2.10_CGAGGCTG	CAAGCAGAAGACGGCATACGAGATCAGCCTCGGTCTCGTGGGCTCGGAGATGT
C03	Ad2.18_GAGGGGTT	CAAGCAGAAGACGGCATACGAGATAACCCCTCGTCTCGTGGGCTCGGAGATGT
D01	Ad2.3_AGGCAGAA	CAAGCAGAAGACGGCATACGAGATTTCTGCCTGTCTCGTGGGCTCGGAGATGT
D02	Ad2.11_AAGAGGCA	CAAGCAGAAGACGGCATACGAGATTGCCTCTTGTCTCGTGGGCTCGGAGATGT
D03	Ad2.19_AGGTTGGG	CAAGCAGAAGACGGCATACGAGATCCCAACCTGTCTCGTGGGCTCGGAGATGT
E01	Ad2.4_TCCTGAGC	CAAGCAGAAGACGGCATACGAGATGCTCAGGAGTCTCGTGGGCTCGGAGATGT
E02	Ad2.12_GTAGAGGA	CAAGCAGAAGACGGCATACGAGATTCCTCTACGTCTCGTGGGCTCGGAGATGT
E03	Ad2.20_GTGTGGTG	CAAGCAGAAGACGGCATACGAGATCACCACACGTCTCGTGGGCTCGGAGATGT
F01	Ad2.5_GGACTCCT	CAAGCAGAAGACGGCATACGAGATAGGAGTCCGTCTCGTGGGCTCGGAGATGT
F02	Ad2.13_GTCGTGAT	CAAGCAGAAGACGGCATACGAGATATCACGACGTCTCGTGGGCTCGGAGATGT
F03	Ad2.21_TGGGTTTC	CAAGCAGAAGACGGCATACGAGATGAAACCCAGTCTCGTGGGCTCGGAGATGT
G01	Ad2.6_TAGGCATG	CAAGCAGAAGACGGCATACGAGATCATGCCTAGTCTCGTGGGCTCGGAGATGT
G02	Ad2.14_ACCACTGT	CAAGCAGAAGACGGCATACGAGATACAGTGGTGTCTCGTGGGCTCGGAGATGT
G03	Ad2.22_TGGTCACA	CAAGCAGAAGACGGCATACGAGATTGTGACCAGTCTCGTGGGCTCGGAGATGT
H01	Ad2.7_CTCTCTAC	CAAGCAGAAGACGGCATACGAGATGTAGAGAGGTCTCGTGGGCTCGGAGATGT
H02	Ad2.15_TGGATCTG	CAAGCAGAAGACGGCATACGAGATCAGATCCAGTCTCGTGGGCTCGGAGATGT
H03	Ad2.23_TTGACCCT	CAAGCAGAAGACGGCATACGAGATAGGGTCAAGTCTCGTGGGCTCGGAGATGT
