# Supplementary material for: Genotyping MUltiplexed-Sequencing of CRISPR-Localized Editing (GMUSCLE): An Experimental and Computational Approach for Analyzing CRISPR-Edited Cells
Source: CRISPR J. 2023 Oct 10;6(5):462–72. doi: 10.1089/crispr.2023.0021 (PMC10611965; doi:10.1089/crispr.2023.0021)
Supplement: Supplemental data [file Supp_TableS3.pdf]

Table S3. List of the 49 major genotypes detected in the 20 samples (in the order of genomic positions).

ID	Genotype (CHR-POS-REF-ALT)	Indel
G1	chr21-34715951-CTATAGTCCAGTACATTGTATAAAGACCACAGG-C	del-32nt
G2	chr21-34715953-ATAGTCCAGTACATTGTATAAAGACCACAGG-A	del-30nt
G3	chr21-34715953-ATAGTCCAGTACATTGTATAAAGACCACAGGTAAGGAAGATG-A	del-41nt
G4	chr21-34715954-TAGTCCAGTACATTGTATAAAGA-T	del-22nt
G5	chr21-34715955-AGTCCAGTACATTGTATAAAGACC-A	del-23nt
G6	chr21-34715958-CCAGTACATTGTATAAAGACCACAGGTA-TATATAGGTGTCTAT	del-13nt
G7	chr21-34715960-AGTACATTGTATAAAGACCACAG-A	del-22nt
G8	chr21-34715960-AGTACATTGTATAAAGACCACAGGTAAGGAAGAT-A	del-33nt
G9	chr21-34715962-TACATTGTATAAAG-T	del-13nt
G10	chr21-34715962-TACATTGTATAAAGACCACAGGTAAGG-T	del-26nt
G11	chr21-34715963-ACATTGTATAAAGA-A	del-13nt
G12	chr21-34715964-CATTGTATAAAGACCACAG-C	del-18nt
G13	chr21-34715964-CATTGTATAAAGACCACAGGTAAGGAAGAT-C	del-29nt
G14	chr21-34715965-ATTGTATAAAGACCACAGGTAAGGAAGATGT-GT	del-29nt
G15	chr21-34715966-TTGTATAAAGACCACAGGTAAGGAAGA-T	del-26nt
G16	chr21-34715967-TGTATAAAGAC-T	del-10nt
G17	chr21-34715967-TGTATAAAGACCACA-T	del-14nt
G18	chr21-34715967-TGTATAAAGACCACAG-T	del-15nt
G19	chr21-34715968-GTATAAAGAC-G	del-9nt
G20	chr21-34715968-GTATAAAGACCACAGGTAAGGAAGATGTTTTGTT-G	del-33nt
G21	chr21-34715969-TATAAAGACCACA-T	del-12nt
G22	chr21-34715970-ATAAAGACCACAGG-A	del-13nt
G23	chr21-34715970-ATAAAGACCACAGGTAAGGAAGATGTTTTGTTTTAGATTCA-T	del-40nt
G24	chr21-34715970-ATAAAGACCACAGGTAAGGAAGATGTTTTGTTTTAGATTCAA-TA	del-40nt
G25	chr21-34715971-TAAAGACCACAGGTA-TA	del-13nt
G26	chr21-34715972-AAAGACCACAGGT-A	del-12nt
G27	chr21-34715972-AAAGACCACAGGTAAGG-A	del-16nt
G28	chr21-34715973-AAGACCACAGGTAA-AA	del-12nt
G29	chr21-34715973-AAGACCACAGGTAAGGAA-AA	del-16nt
G30	chr21-34715974-AGACCACAGGTAAG-A	del-13nt
G31	chr21-34715974-AGACCACAGGTAAGGAAGATGTTTT-A	del-24nt
G32	chr21-34715975-GAC-G	del-2nt
G33	chr21-34715975-GACC-A	del-3nt
G34	chr21-34715975-GACC-G	del-3nt
G35	chr21-34715975-GACCACAGGT-G	del-9nt
G36	chr21-34715976-AC-A	del-1nt
G37	chr21-34715976-ACC-T	del-2nt
G38	chr21-34715976-ACCACAGG-ATGTA	del-3nt
G39	chr21-34715976-ACCACAGGTA-ATGTG	del-5nt
G40	chr21-34715977-CCA-C	del-2nt
G41	chr21-34715977-CCACAG-C	del-5nt
G42	chr21-34715977-CCACAGGT-C	del-7nt
G43	chr21-34715977-CCACAGGTAAGGAAGA-C	del-15nt
G44	chr21-34715977-CCACAGGTAAGGAAGAT-C	del-16nt
G45	chr21-34715977-CCACAGGTAAGGAAGATGTT-C	del-19nt
G46	chr21-34715978-CACA-AG	del-2nt
G47	chr21-34715978-CACAG-AAT	del-2nt
G48	chr21-34715975-GACC-TGTACTT	ins-3nt
G49	chr21-34715978-CACAGG-AGGTAAGGAAGATGTATAAAGTGTA	ins-19nt
