# Supplementary material for: Genotyping MUltiplexed-Sequencing of CRISPR-Localized Editing (GMUSCLE): An Experimental and Computational Approach for Analyzing CRISPR-Edited Cells
Source: CRISPR J. 2023 Oct 10;6(5):462–72. doi: 10.1089/crispr.2023.0021 (PMC10611965; doi:10.1089/crispr.2023.0021)
Supplement: Supplemental data [file Supp_TableS4.pdf]

Table S4. Matrix of 49 major genotypes (G1-G49) from 20 samples (S1-S20). Genotype ID is as in Table S3.

ID	S1	S2	S3	S4	S5	S6	S7	S8	S9	S10	S11	S12	S13	S14	S15	S16	S17	S18	S19	S20
G1	.	.	.	.	.	.	.	.	.	.	.	.	26%	.	.	.	.	.	.	.
G2	.	.	.	.	.	.	.	.	.	.	.	.	.	21%	.	.	.	.	.	.
G3	.	.	.	.	.	.	.	.	.	.	.	.	.	.	22%	.	.	.	.	.
G4	.	.	.	.	.	.	20%	.	.	.	.	.	.	.	.	.	.	.	.	.
G5	.	.	19%	.	.	.	.	.	.	.	.	.	.	.	.	.	.	.	.	.
G6	.	.	.	.	.	.	.	.	.	.	.	.	.	.	.	.	19%	.	.	.
G7	.	.	.	.	.	.	.	.	.	18%	.	.	.	.	.	.	.	.	.	.
G8	21%	.	.	.	.	.	.	.	.	.	.	.	.	.	.	.	.	.	.	.
G9	.	.	.	.	.	.	.	.	.	.	.	.	.	.	.	32%	.	.	.	.
G10	.	.	.	.	.	.	.	.	.	.	.	.	.	21%	.	.	.	.	.	.
G11	.	.	.	.	.	.	.	.	.	.	.	.	.	.	.	1%	.	.	.	.
G12	.	.	.	.	.	.	.	15%	.	.	.	.	.	.	.	.	.	.	20%	.
G13	.	.	.	.	.	11%	.	.	.	.	.	.	.	.	.	.	.	.	.	.
G14	.	.	.	.	.	2%	.	.	.	.	.	.	.	.	.	.	.	.	.	.
G15	.	.	.	.	.	.	19%	.	.	.	.	18%	.	.	.	.	.	.	.	.
G16	.	.	.	.	.	.	21%	.	.	.	.	.	.	.	.	.	.	.	.	.
G17	.	.	17%	.	.	.	.	.	.	.	.	.	.	.	.	.	.	.	.	.
G18	.	20%	.	.	.	.	.	.	.	.	.	.	.	.	.	.	23%	.	.	30%
G19	.	.	16%	.	.	.	.	.	.	.	.	.	.	.	.	.	.	.	.	.
G20	.	.	.	.	.	.	.	.	.	.	.	11%	.	.	.	.	.	.	.	.
G21	.	.	.	.	.	.	.	.	.	.	.	.	.	.	.	.	.	19%	.	.
G22	.	.	.	.	.	7%	.	13%	17%	.	.	.	.	.	20%	.	.	21%	.	.
G23	.	.	.	.	.	.	.	.	.	.	.	.	34%	.	.	.	.	.	.	.
G24	.	.	.	.	.	.	.	.	.	.	.	.	1%	.	.	.	.	.	.	.
G25	.	.	.	.	.	1%	.	.	.	.	.	.	.	.	.	.	.	.	.	.
G26	.	.	.	.	.	7%	.	.	.	.	.	.	.	.	.	.	.	20%	.	.
G27	.	.	.	.	.	11%	.	.	.	.	.	.	.	.	.	.	.	.	.	.
G28	.	.	.	.	.	8%	.	.	.	.	.	.	.	.	.	.	.	.	.	.
G29	.	.	.	.	.	2%	.	.	.	.	.	.	.	.	.	.	.	.	.	.
G30	.	19%	.	.	.	.	.	.	.	.	.	.	.	.	.	.	.	.	.	.
G31	.	.	.	.	.	.	.	.	.	.	.	.	.	.	.	.	.	.	20%	.
G32	.	.	.	.	.	.	.	.	.	.	.	.	.	.	.	.	.	.	.	30%
G33	.	.	.	16%	.	.	.	.	.	.	.	.	.	.	.	.	.	.	.	.
G34	14%	21%	.	.	.	5%	.	.	.	15%	17%	.	.	17%	19%	.	.	.	.	.
G35	.	.	.	.	.	.	.	.	17%	.	.	.	.	.	.	.	.	.	.	.
G36	.	.	.	16%	.	.	.	15%	.	.	15%	.	.	.	.	.	.	.	19%	.
G37	13%	.	.	.	.	.	.	.	.	.	.	.	.	.	.	.	.	.	.	.
G38	.	.	.	.	.	.	.	.	.	.	.	10%	.	.	.	.	.	.	.	.
G39	.	.	.	.	.	.	.	.	.	.	.	.	.	.	.	29%	.	.	.	.
G40	.	.	.	.	33%	.	.	.	.	13%	.	.	.	.	.	.	.	.	.	.
G41	13%	.	.	.	.	.	.	.	.	.	16%	.	.	.	.	.	.	.	.	.
G42	.	.	.	.	.	6%	.	.	.	.	.	.	.	.	.	.	.	.	.	.
G43	.	.	.	.	30%	.	.	.	.	.	.	.	.	.	.	.	.	.	.	.
G44	.	.	.	.	.	.	.	.	.	13%	.	.	.	.	.	.	.	.	.	.
G45	.	.	.	14%	.	.	.	.	.	.	.	.	.	.	.	.	.	.	.	.
G46	.	.	.	.	.	.	.	16%	.	.	.	.	.	.	.	.	.	.	.	.
G47	.	.	.	.	.	.	.	.	.	.	17%	.	.	.	.	.	.	.	.	.
G48	.	.	.	.	.	.	.	.	.	.	.	.	.	.	.	.	21%	.	.	.
G49	.	.	.	14%	.	.	.	.	.	.	.	.	.	.	.	.	.	.	.	.
