# Supplementary material for: Genotyping MUltiplexed-Sequencing of CRISPR-Localized Editing (GMUSCLE): An Experimental and Computational Approach for Analyzing CRISPR-Edited Cells
Source: CRISPR J. 2023 Oct 10;6(5):462–72. doi: 10.1089/crispr.2023.0021 (PMC10611965; doi:10.1089/crispr.2023.0021)

Supplementary Figures

Figure S1. The read count of the top 100 unique reads in each sample (S1-S10). The y-axis of read count is capped and fixed at 1,000, in order to visualize the reads of low counts. The exact read counts of the top unique reads are given in Table S1.

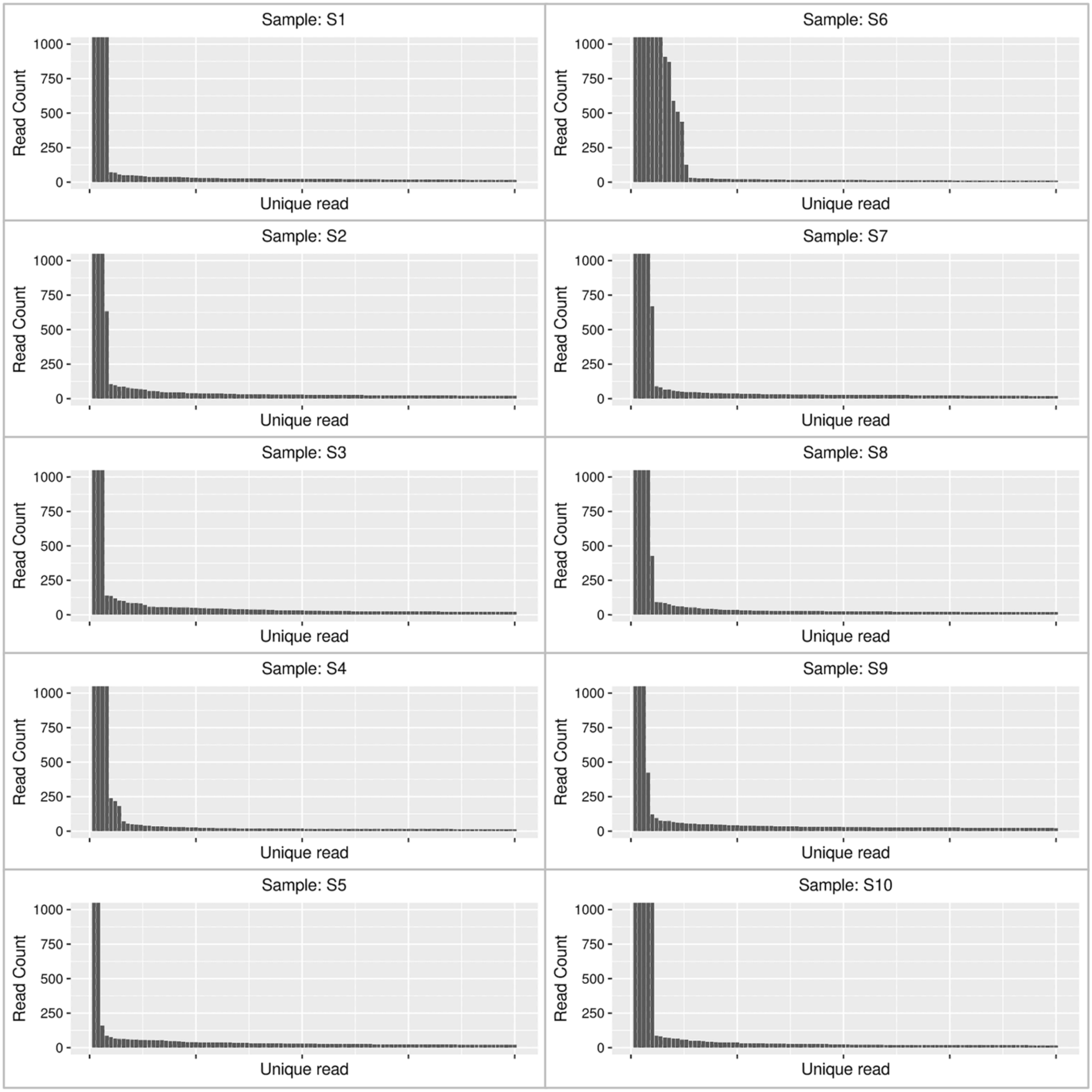

Figure S1. (continued) **The read count of the top 100 unique reads in each sample (S11-S20).** The y-axis of read count is capped and fixed at 1,000, in order to visualize the reads of low counts. The exact read counts of the top unique reads are given in Table S1.

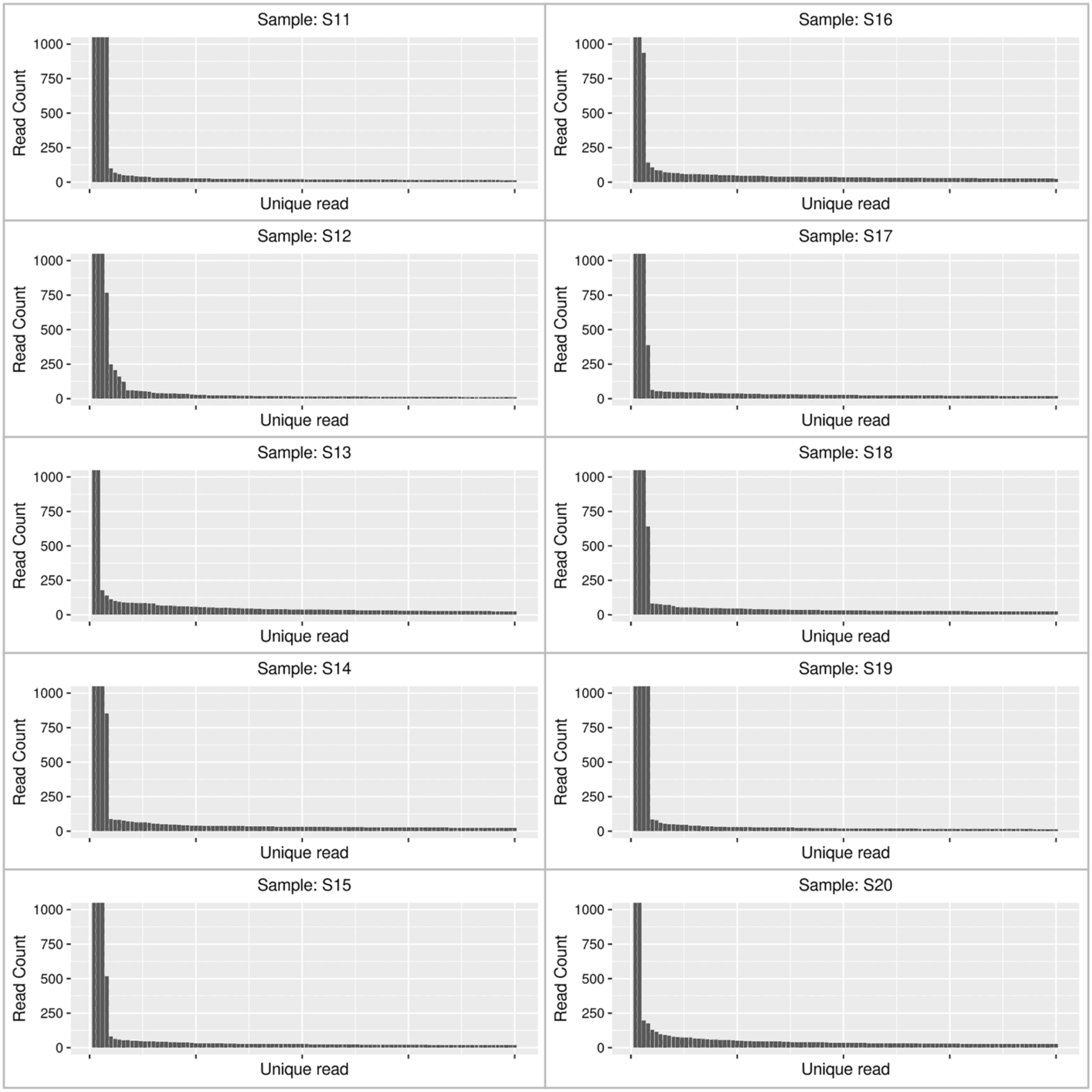

Supplement: Supplemental data [file Supp_FigS1.pdf]
