# Supplementary material for: Genotyping MUltiplexed-Sequencing of CRISPR-Localized Editing (GMUSCLE): An Experimental and Computational Approach for Analyzing CRISPR-Edited Cells
Source: CRISPR J. 2023 Oct 10;6(5):462–72. doi: 10.1089/crispr.2023.0021 (PMC10611965; doi:10.1089/crispr.2023.0021)
Supplement: Supplemental data [file Supp_TableS2.pdf]

Table S2. The read count of the top-20 unique reads in the 20 samples.

Top-20 unique reads	S1	S2	S3	S4	S5	S6	S7	S8	S9	S10
#1	11,404	13,519	12,814	8,318	17,657	6,213	14,359	10,436	19,410	9,097
#2	7,535	13,442	11,468	8,047	16,387	6,041	12,100	9,939	11,144	8,526
#3	7,333	11,814	10,882	7,287	160	4,186	10,270	9,565	10,991	8,018
#4	7,158	634	138	7,283	87	3,608	2,959	8,042	425	7,985
#5	71	105	136	238	76	3,447	671	427	120	2,506
#6	68	98	117	217	67	3,371	90	93	95	87
#7	56	88	102	183	64	2,900	81	89	76	82
#8	50	86	100	71	63	907	66	83	74	75
#9	49	78	88	54	61	872	65	77	73	71
#10	49	73	83	51	59	588	58	65	66	66
#11	48	72	83	48	58	510	54	61	61	65
#12	45	68	82	45	55	437	50	61	60	58
#13	43	67	71	39	55	126	48	55	55	58
#14	38	55	59	39	54	32	47	54	53	51
#15	38	55	58	35	54	30	47	52	52	51
#16	38	54	56	34	52	27	44	48	51	50
#17	38	47	55	34	52	27	43	43	50	47
#18	37	46	55	33	49	26	42	43	50	43
#19	37	45	55	31	48	26	40	42	49	42
#20	37	45	54	30	47	25	40	41	48	39
**Top-20 unique reads**	**S11**	**S12**	**S13**	**S14**	**S15**	**S16**	**S17**	**S18**	**S19**	**S20**
#1	10,248	8,594	20,297	14,635	12,376	19,555	13,480	13,116	8,491	18,729
#2	10,135	5,884	15,577	14,125	10,836	18,087	11,606	12,989	8,300	18,679
#3	9,372	5,058	179	11,840	10,538	937	10,603	12,480	5,954	196
#4	9,251	767	138	853	519	143	388	642	2,723	176
#5	100	248	113	86	82	108	64	82	85	129
#6	69	206	101	82	64	86	53	79	78	115
#7	57	159	94	81	58	84	53	76	60	97
#8	50	123	89	76	54	72	51	72	53	93
#9	48	62	88	71	54	69	51	70	51	86
#10	47	61	87	69	51	67	48	63	50	79
#11	43	59	85	64	51	67	47	55	47	77
#12	40	55	84	63	47	60	47	54	46	75
#13	40	52	83	63	46	59	46	53	45	73
#14	37	49	82	60	45	59	45	52	41	73
#15	32	42	81	54	45	57	44	52	40	65
#16	32	40	68	54	43	57	44	51	39	65
#17	32	39	67	50	43	56	42	50	35	63
#18	32	38	65	49	42	55	41	48	34	62
#19	31	36	65	48	41	53	40	48	34	59
#20	30	36	64	47	39	53	39	47	33	57
