# Supplementary material for: Genotyping MUltiplexed-Sequencing of CRISPR-Localized Editing (GMUSCLE): An Experimental and Computational Approach for Analyzing CRISPR-Edited Cells
Source: CRISPR J. 2023 Oct 10;6(5):462–72. doi: 10.1089/crispr.2023.0021 (PMC10611965; doi:10.1089/crispr.2023.0021)

Figure S2. Genotype plots generated for each sample (S1-S10).

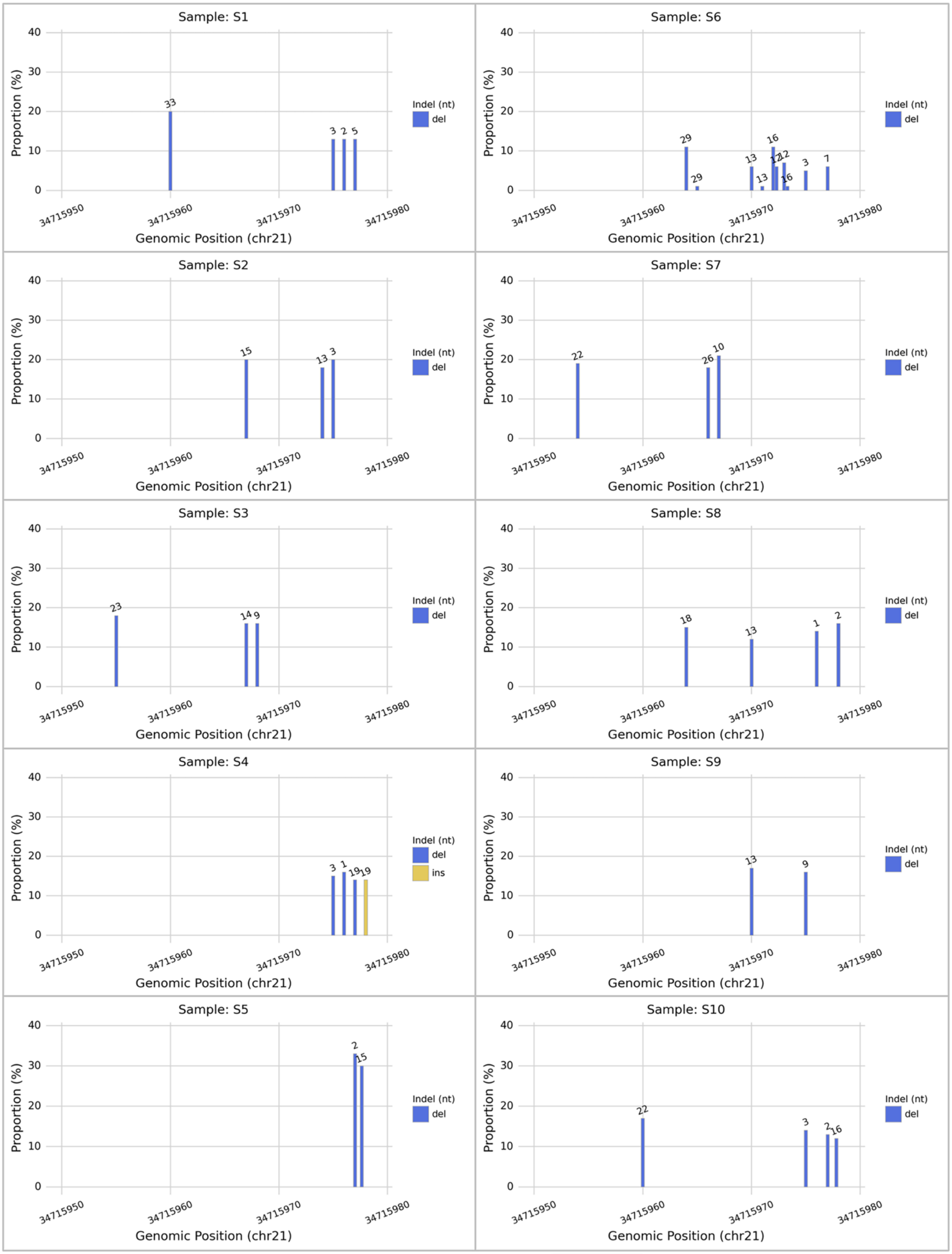

Figure S2. (continued) **Genotype plots generated for each sample (S11-S20).**

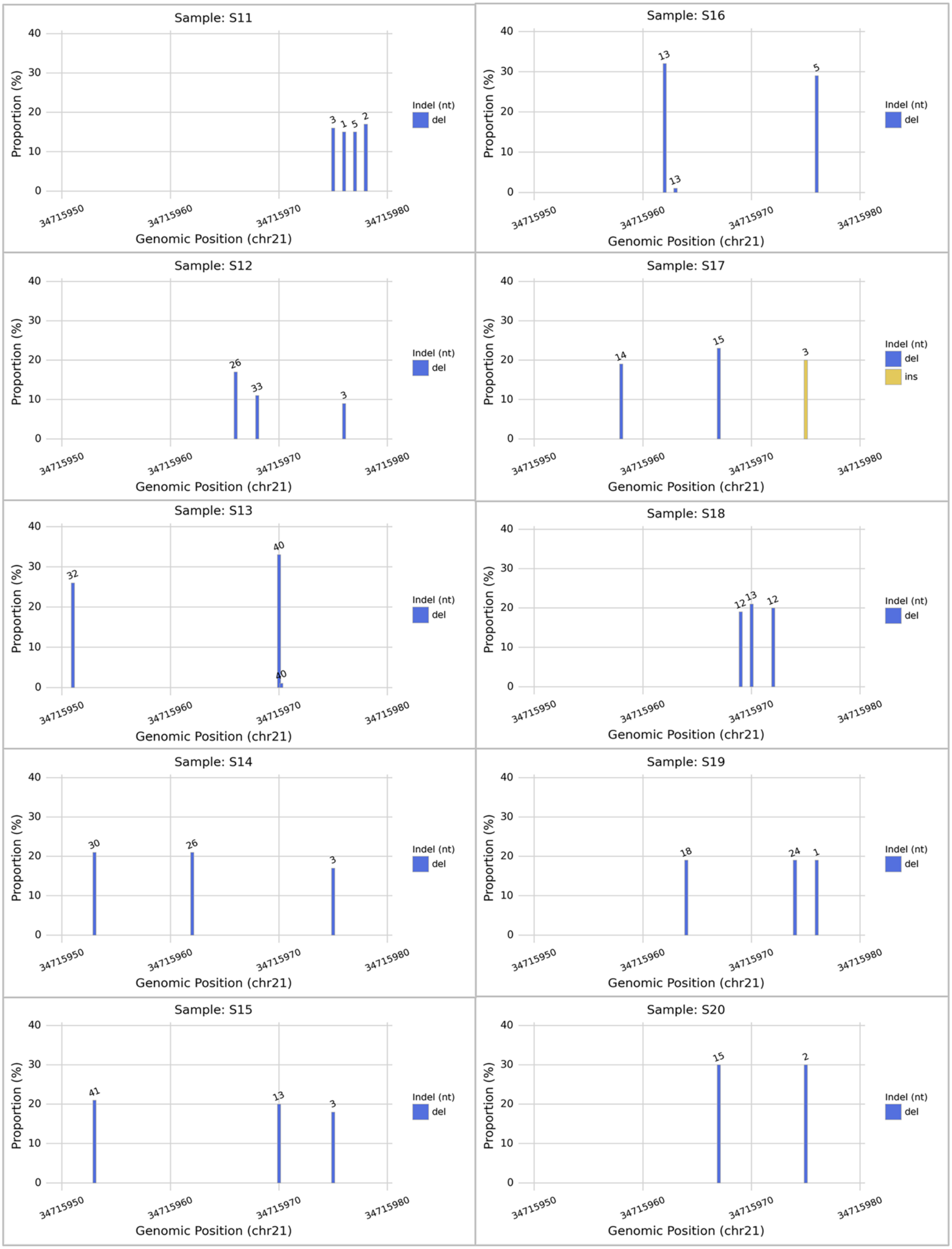

Supplement: Supplemental data [file Supp_FigS2.pdf]
