# Supplementary material for: Genotyping MUltiplexed-Sequencing of CRISPR-Localized Editing (GMUSCLE): An Experimental and Computational Approach for Analyzing CRISPR-Edited Cells
Source: CRISPR J. 2023 Oct 10;6(5):462–72. doi: 10.1089/crispr.2023.0021 (PMC10611965; doi:10.1089/crispr.2023.0021)
Supplement: Supplemental data [file Supp_TableS5.pdf]

Table S5. The use of read-count cutoff to retain unique reads for the identification of major genotypes. Sample S1 was used as the example for demonstration, and the use of the various read-count cutoffs (from 0 to 50) invariably led to identification of the same four major genotypes.

**Read-count cutoff**	0	1	5	10	30	50
**No. of reads retained for genotyping**	56,269	43,790	40,060	37,474	34,340	33,625
**% reads retained from total reads**	100%	77.8%	71.2%	66.6%	61.0%	59.8%
**No. of unique reads**	14,336	1,856	566	217	24	6
**No. of total genotypes**	832	294	137	58	6	4
**No. of major genotypes (≥1% of total reads)**	4	4	4	4	4	4
**% reads supporting genotype_1**	24.7%	24.1%	22.9%	22.1%	20.8%	20.4%
**% reads supporting genotype_2**	16.5%	15.9%	15.0%	14.3%	13.7%	13.4%
**% reads supporting genotype_3**	16.0%	15.5%	14.6%	14.1%	13.3%	13.0%
**% reads supporting genotype_4**	15.8%	15.2%	14.4%	13.8%	13.1%	13.0%
**% reads supporting major genotypes**	73.0%	70.7%	66.9%	64.3%	60.9%	59.8%
**Read contribution to major genotypes**	0.73	0.91	0.94	0.97	1.00	1.00
**Time cost (seconds)**	32	12	11	10	10	10

("Read-count cutoff" is based on the counts of each unique read in the input fastq file; "major genotype" is defined as a genotype supported by ≥1% of total reads; "read contribution to major genotypes" is defined as "the number of reads supporting major genotypes" divided by "the number of reads retained from total reads", indicating the proportion of the retained reads supporting the major genotypes.)
